# The volatilization behaviour of typical fluorine-containing slag in steelmaking

**DOI:** 10.1098/rsos.200704

**Published:** 2020-08-26

**Authors:** Zhongyu Zhao, Junxue Zhao, Zexin Tan, Boqiao Qu, Yaru Cui

**Affiliations:** School of Metallurgical Engineering, Xi'an University of Architecture and Technology, 13 Yanta Road, Xi'an, Shaanxi 710055. People's Republic of China

**Keywords:** fluorine-containing slag, volatilization, thermal analysis, steelmaking

## Abstract

It was taken as typical steelmaking fluorine-containing slag systems with the remelting electroslag, continuous casting mould flux and refining slag. The volatilization behaviour of each slag system was analysed by thermogravimetric (TG) and mass spectrometry (MS) detection. The results showed that the remelting electroslag volatilized significantly above 1300°C and the volatiles were mainly CaF_2_, MgF_2_ with a small amount of SiF_4_ and AlF_3_; the continuous casting mould flux volatilization was divided into two stages, in the first stage (500°C∼800°C), CaF_2_ and Na_2_O reacted to form NaF, and in the second stage (greater than 1200°C), the CaF_2_ evaporation was highlighted; for CaF_2_-CaO-based refining slag, the volatilization was the most significant at the eutectic point 84% CaF_2_–16% CaO, and the volatility can be reduced by adding 5% SiO_2_. This research will be guiding significance for the composition and performance control of fluorine-containing slag and metallurgical environmental protection in the steelmaking process.

## 1. Introduction

The steelmaking process is actually a ‘slag-making’ process. The physico-chemical properties of slag are of significant effects on the melting temperature, chemical reactions, metal solidification and inclusions removal. Therefore, the slag composition must be reasonably controlled to meet different melting requirements [1]. The fluoride and potassium sodium oxides are widely used as fluxes to satisfy the slag high-temperature physico-chemical properties, and the form and amount of fluoride depend on the production indexes of different steel plants and the performance requirements of different steel. It is generally known that the steelmaking process is mostly in heating or holding process to maintain the slag–metal reaction. The slag will volatilize at high temperature and eventually change the slag composition and metallurgical properties, if the slag contains fluoride or alkali metal oxide (Na_2_O, K_2_O) [2–12].

On these issues, a large number of scholars have done some research on the slag volatilization. Mills [13] and Mao [2] judged that the volatiles were NaF, KF, SiF_4_, AlF_3_ and CaF_2_ by thermodynamic calculation. Chen and Liang [14–17] simulated the fluoride volatilization process by establishing kinetic models. Zhao *et al.* [18] analysed the important influence of fluoride on the properties of mould flux, such as melting point, viscosity and crystalline. Shang *et al.* [19] summarized the influence of slag volatilization characteristics on the physico-chemical properties by measuring the melting point and viscosity of fluorine-containing electroslag. However, the study around volatiles is still limited to the theoretical calculation or indirect experimental analysis, and the conclusions are not convincing. The qualitative and quantitative analysis is insufficient. In this paper, three kinds of typical fluorine-containing slag systems for steelmaking process will be investigated and compared with the volatilization characteristics by thermogravimetric (TG), mass spectrometry (MS) and X-ray fluorescence (XRF) methods, which can directly reflect the volatilization characteristics of different steelmaking slag. It can be practical and application value of slag composition and metallurgical performance control in steelmaking.

## 2. Materials and methods

**Figure 1.**
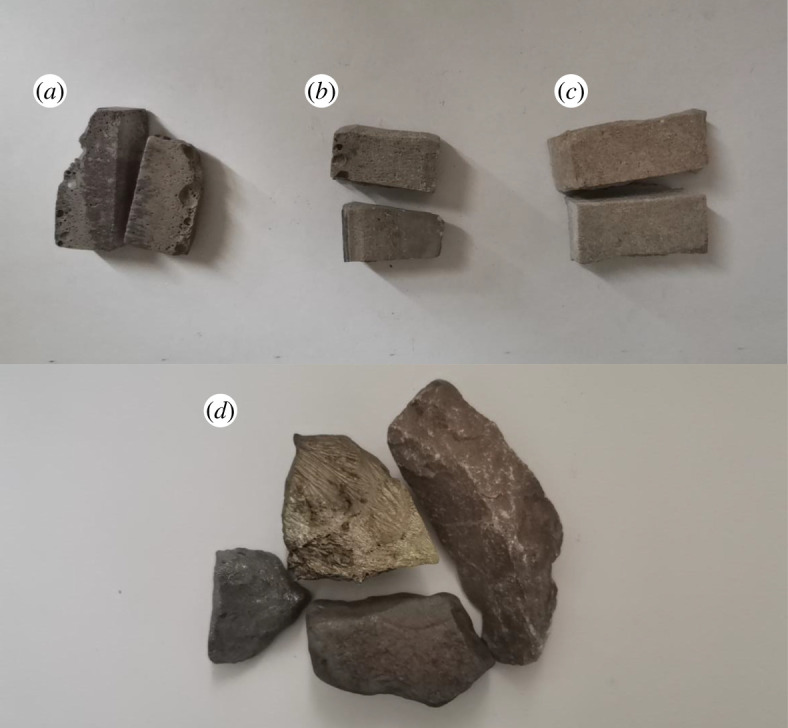
Fluorine-containing slag. (*a*) Remelting electroslag, E1; (*b*) remelting electroslag, E2, E3; (*c*) remelting electroslag, E4; (*d*) continuous casting mould flux.

In order to systematically analyse the slag volatilization behaviour in the steelmaking process, three typical steelmaking fluorine-containing slag systems were selected from Taiyuan Iron&Steel (Group) Co., Ltd, of which the annual production capacity is 0.3 million tons of steel ingots, including the remelting electroslag, the continuous casting mould flux and the traditional refining slag based on CaF_2_-CaO, shown in figure 1.

The volatilization characteristics of each slag system were analysed by TG-MS. The specific research methods were described as follows:

— Take XRF-1800 by melt press in Pt-Rh crucible to detect the slag composition and prepare different slag samples with chemical reagents according to XRF results. The samples were ground by an agate ball mill at a speed of 200 r.p.m. for 0.5 h, dried at 373 K for 5 h and then sealed and stored in the dark.
— For the remelting electroslag and continuous casting mould flux, take TG-MS tests to determine the volatilization temperature, different volatiles and volatilization ratio (take NETZSCH 449-F3 analyser for TG test and STA 409 C/CD mass spectrometer for MS test); for CaF_2_-CaO-based refining slag, it was prepared with chemical pure reagents according to different proportions. The reagent information is shown in table 1. The weight loss of each sample was recorded by constant temperature heating in a tubular furnace. The experimental parameters were set as follows:
  (i) TG test: the heating rate was 10°C min^−1^ with Ar gas at a flow rate of 50 ml min^−1^;
  (ii) MS test: the heating rate was 10°C min^−1^ (stage I); the temperature was maintained for 1 h at 1300°C (stage II) and then increased to 1400°C (stage III). Finally, the sample was slowly cooled (stage IV). It was protected with Ar gas at a flow rate of 50 ml min^−1^.
  (iii) Tubular furnace heating process: the refining slag samples mixed with reagents were prepared according to different proportions (CaF_2_ pure reagent, 90% CaF_2_–10% CaO, 84% CaF_2_–16% CaO, 60% CaF_2_–40% CaO, 40% CaF_2_–60% CaO); tube furnace was set at 1500°C and samples were weighed within 5–30 min with Ar gas protection at a flow rate of 50 ml min^−1^.
    (Note: the platinum crucible was used in the TG-MS test and the graphite crucible was used in the tube furnace heating process.)
— The components of roasted samples were analysed by XRF for testing the above discussion on the volatilization characteristics of each slag system.

**Table 1.** The reagent information.

reagent	purity %	granularity	batch
CaF_2_	≥98.5	white powder	20160109
CaO	≥98.0	white powder	20160807
SiO_2_	≥99.0	(0.65 ∼ 0.85 mm) ≥ 89.0%	20160819
Al_2_O_3_ (neutral)	>99.0	white powder	20160814
MgO (light)	≥98.5	white powder	20160709
Na_2_CO_3_	≥99.8	white powder	20161022

## 3. Results and discussion

### 3.1. Volatilization behaviour of remelting electroslag

**Figure 2.**
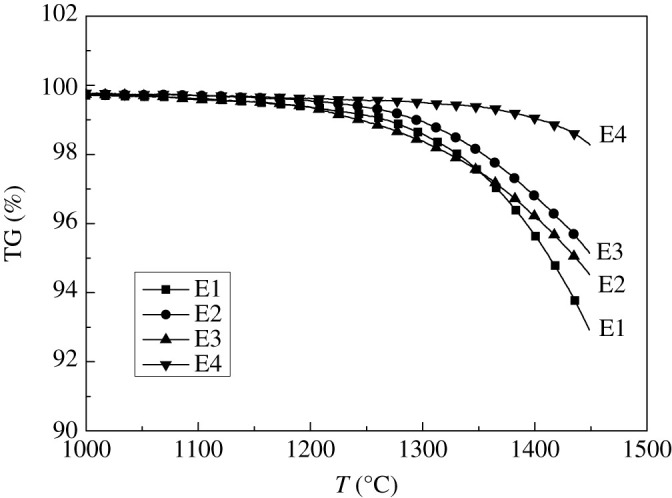
TG curves of remelting electroslag system.

Four kinds of remelting electroslag were analysed by XRF, as shown in table 2. The TG tests were carried out respectively, as shown in figure 2.

**Table 2.** Components of remelting electroslag and the weight loss in TG test (%).

no.	CaF_2_	CaO	SiO_2_	Al_2_O_3_	MgO	weight loss at 1450°C
E1	39.64	24.82	10.25	18.31	6.98	7.1
E2	31.52	32.05	11.65	17.66	7.12	4.8
E3	32.06	24.59	19.15	17.15	7.05	5.5
E4	27.35	35.06	9.64	17.93	10.02	1.8

According to the weight loss curves in figure 2, the electroslag with different fluorine content had different weight loss, and the more the fluorine, the greater the weight loss rates. When the temperature was higher than 1300°C, the weight loss was obvious. It can be seen that the volatilization characteristics of remelting electroslag were directly related to CaF_2_ and temperature.

**Figure 3.**
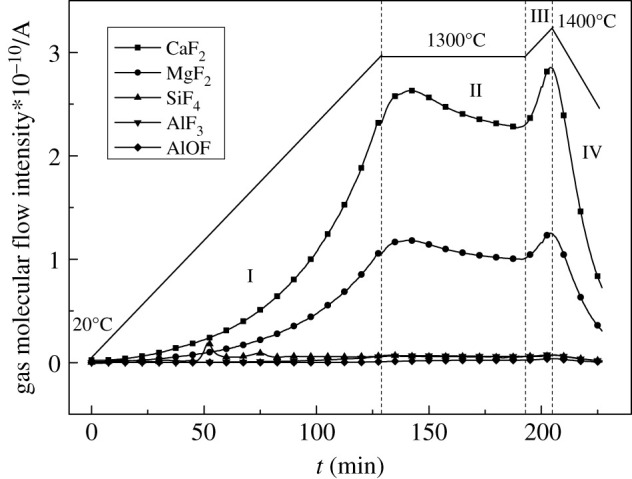
MS curves of remelting electroslag system (E3).

To further explore the volatiles, the E3 sample was tested by MS and the results are shown in figure 3.

It can be seen from figure 3 that during the initial volatilization stage I (750°C–1200°C), the volatiles were divided into a small amount of MgF_2_, SiF_4_ and AlF_3_, and from beginning to end, especially in high-temperature regions II–III (1300°C–1400°C), CaF_2_ was the main volatile. This was basically consistent with the previous thermodynamic calculation results [14].

### 3.2. Volatilization behaviour of continuous casting mould flux

**Figure 4.**
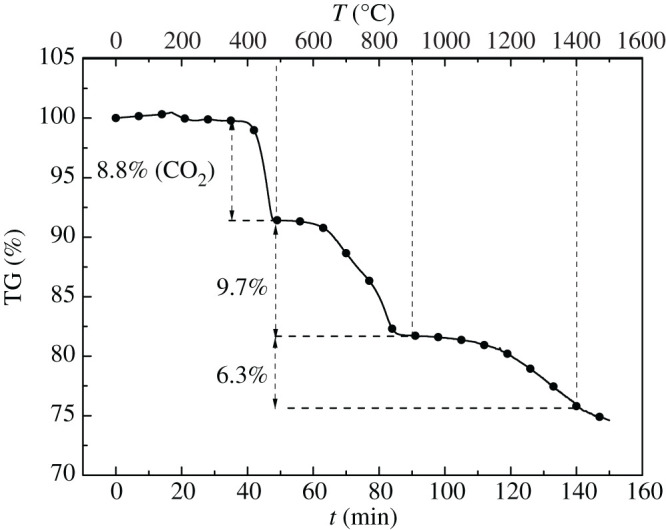
TG curve of the continuous casting mould flux. (Note: the weight loss before 500°C could be ignored considering the decomposition reaction of Na_2_CO_3_, as follows. Na_2_CO_3_ ≜ Na_2_O + CO_2_↑).

The continuous casting mould flux was mixed by chemical pure reagents and its composition is shown in table 3. The TG test is shown in figure 4.

**Table 3.** Composition of the continuous casting mould flux (%).

composition	CaF_2_	Al_2_O_3_	MgO	SiO_2_	CaO	Na_2_CO_3_	weight loss at 1400°C
quality	17.6	3.8	3.0	27.2	27.2	21.2	16.0

**Figure 5.**
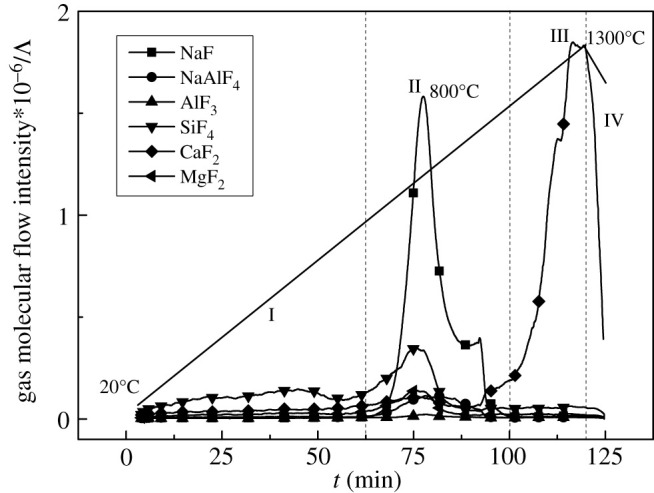
MS curves of the continuous casting mould flux.

According to TG curve in figure 4, the weight loss process could be divided into two stages: the first stage was from 500°C to 900°C and the weight loss rate was 9.7%; the second stage was above 1000°C, and the weight loss rate was 6.3%. According to the previous thermodynamic calculation under the same experimental conditions [18], the first stage was the reaction of CaF_2_ with Na_2_O and SiO_2_ to generate NaF and SiF_4_ gas, and the second stage was mainly the CaF_2_ evaporation, as shown in table 4. To determine the volatiles of the above samples, the MS test was carried out and is shown in figure 5.

**Table 4.** Volatiles reaction.

no.	reaction
(1)	CaF_2_(s) = CaF_2_(g)↑
(2)	CaF_2_(s) + 1/2SiO_2_(s) = 1/2SiF_4_(g)↑ + CaO(s)
(3)	CaF_2_(s) + MgO(s) = MgF_2_(g)↑ + CaO(s)
(4)	CaF_2_(s) + 1/3Al_2_O_3_(s) = CaO(s) + 10AlF_3_(g)↑
(5)	CaF_2_(s) + Na_2_O(s) = 2NaF (g)↑ + CaO (g)

From the MS curves in figure 5, it can be seen that the volatilization of continuous casting mould flux system was more complex than that of remelting electroslag. Although the gases such as SiF_4_ and MgF_2_ were generated at stage I from 500°C to 800°C, the NaF was volatilized preferentially and largely at stage II due to the strong activity of light metal oxide Na_2_O. Similarly, when it was above 1200°C at stage III, the CaF_2_ evaporation was highlighted.

### 3.3. Volatilization behaviour of CaF_2_-CaO-based refining slag

The refining slag played a significant role in the steelmaking process for desulfurization and alloying. Different kinds of steel have different requirements for the properties and composition of refining slag. The traditional refining slag system is based on CaF_2_-CaO, and sometimes added with an appropriate amount of SiO_2_ (4%–11%) and Al_2_O_3_ (6%–9%). Therefore, focus on the volatilization behaviour, the CaF_2_-CaO slag system was prepared with chemical reagents by different proportion and it could reveal the volatilization characteristics of traditional refining slag system.

**Figure 6.**
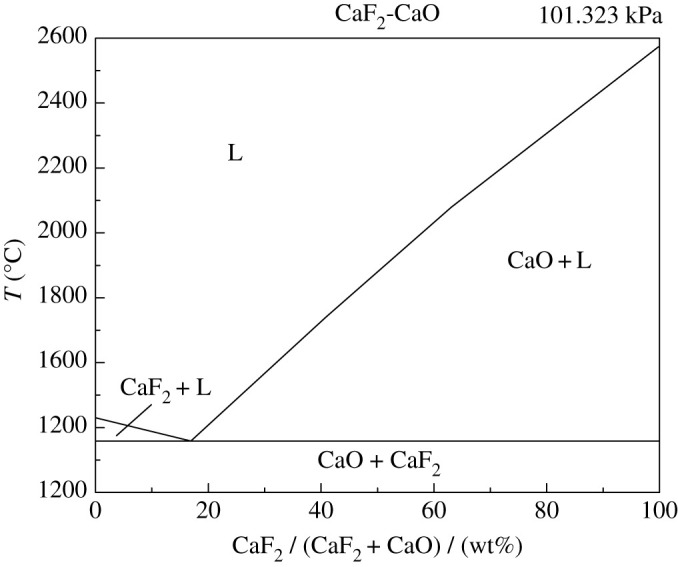
CaF_2_-CaO phase diagram.

**Figure 7.**
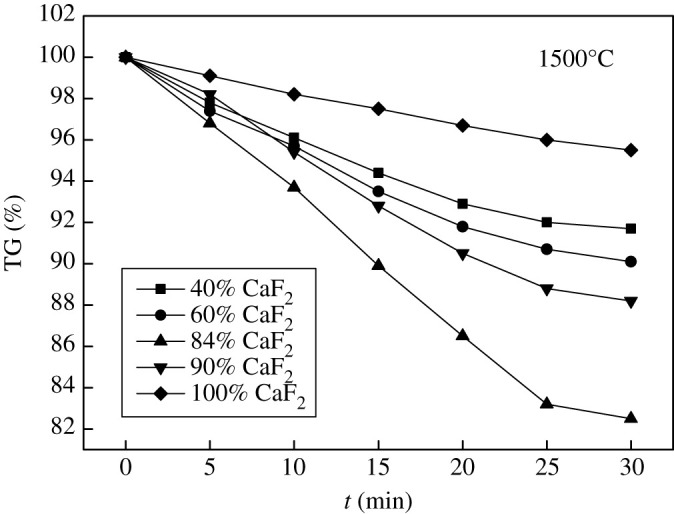
Effect of CaF_2_ on volatilization of refining slag.

Firstly, the binary phase diagram of CaF_2_-CaO was calculated by Factsage, as shown in figure 6, the eutectic point was 84% CaF_2_–16% CaO. Then, the CaF_2_-CaO-based refining slag with different composition was prepared by chemical reagents and heated in a tubular furnace at 1500°C. The weight loss of each sample after different holding time is shown in figure 7.

**Figure 8.**
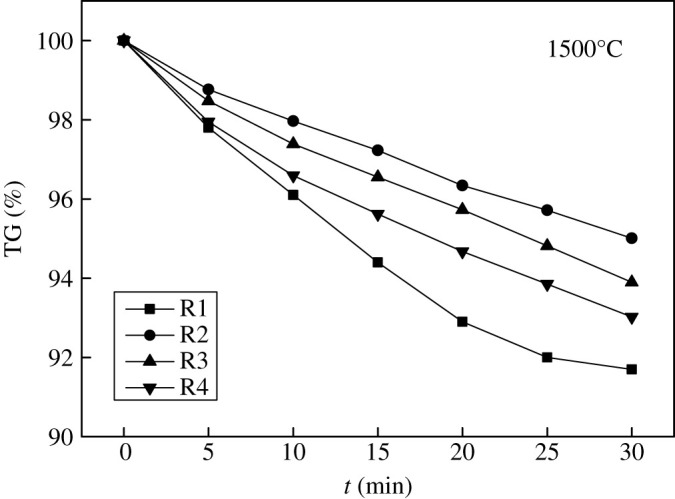
Effect of SiO_2_ on volatilization of refining slag. (R1: 40% CaF_2_–60% CaO; R2: 38% CaF_2_–57% CaO–5% SiO_2_; R3: 36% CaF_2_–54% CaO–10% SiO_2_; R4: 33% CaF_2_–50% CaO–17% SiO_2_).

From figure 7 TG curves, it can be obtained that the volatilization of CaF_2_-CaO slag was of the most significant at the eutectic point 84% CaF_2_–16% CaO, and the volatile was CaF_2_. Therefore, for this refining slag system, the CaF_2_ proportion can be adjusted properly to weaken the slag volatilization. Furthermore, a small amount of SiO_2_ (5%∼10%) was added to the CaF_2_-CaO slag system to explore the effect of SiO_2_ on volatilization at 1500°C, as shown in figure 8.

It can be seen from figure 8 that the volatility of the refining slag system can be reduced by adding a small amount of SiO_2_ comparing R1 and R2. However, the SiF_4_ gas would be generated to accelerate the volatility of the slag system if the SiO_2_ was too much comparing R2, R3 and R4.

### 3.4. Examination of volatilization characteristics

As mentioned above, for the CaF_2_-CaO-based refining slag, the volatile was single CaF_2_, and no further inspection was required. For remelting electroslag and continuous casting mould flux, it was necessary to do XRF analysis of the samples after TG tests due to the complex volatilization characteristics, and this was also in contrast with the results of the above TG-MS analysis. The XRF tests of remelting electroslag and continuous casting mould flux are shown in Tables 5 and 6.

**Table 5.** Components of remelting electroslag after TG tests (%).

no.	CaF_2_	CaO	SiO_2_	Al_2_O_3_	MgO
E1-R	37.33	28.26	9.91	18.77	5.73
E2-R	28.36	34.35	11.55	18.91	6.83
E3-R	27.66	27.54	19.22	19.01	6.57
E4-R	25.58	35.92	10.18	18.33	9.98

**Table 6.** Components of mould flux after TG tests (%).

composition	CaF_2_	Al_2_O_3_	MgO	SiO_2_	CaO	Na_2_O
quality	4.71	5.04	3.32	34.10	46.76	6.06

From the comparison of the results in the above tables with the original composition (Tables 1 and 2), it can be seen that, for the remelted electroslag, the CaF_2_ and MgO were reduced and the CaO was increased. It was basically consistent with the results of TG-MS analysis and the main volatiles were CaF_2_ and MgF_2_. For continuous casting mould flux, in addition to CaF_2_, the Na_2_O also obviously reduced. It was consistent with the TG-MS results, and in addition to the reaction between CaF_2_ and Na_2_O, it was found that a small amount of Na_2_O was evaporated by calculating the Na_2_O weight loss.

The volatilization of fluorine-containing slag in steelmaking is not only difficult to achieve the technical requirements, but also has an impact on the environment considering the toxicity of fluoride. Therefore, it was proposed with the premelted process for fluorine-containing slag [19] that preheat the slag to 1000–1200°C with closed electromagnetic stirring, and then directly participate in the metallurgical process, which can reduce the heat loss, inhibit the volatility of slag and reduce the harm to the environment. The similar effect can also be achieved for the cooled premelted slag after crushing for steelmaking.

## 4. Conclusion

— As typical fluorine-containing slag systems for steelmaking, the remelting electroslag, continuous casting mould flux, and CaF_2_-CaO-based refining slag can be shown with different volatilization characteristics at high temperature.
— The remelting electroslag volatilized significantly above 1300°C and the volatiles were mainly CaF_2_, MgF_2_ with a small amount of SiF_4_ and AlF_3_; the continuous casting mould flux volatilization was divided into two stages, in the first stage (500°C∼800°C), CaF_2_ and Na_2_O reacted to form NaF, and in the second stage (greater than 1200°C), it was mainly the CaF_2_ evaporation; for CaF_2_-CaO-based refining slag, the volatilization was of the most significant at the eutectic point 84% CaF_2_–16% CaO, and the volatility can be reduced by adding 5% SiO_2_.
— Take XRF tests of remelting electroslag and continuous casting mould flux after TG tests, and the results were basically consistent with the previous TG-MS analysis. Therefore, this study will be of significance for both slag composition and volatilization characteristics control and metallurgical environmental protection.

## Supplementary Material

Supplementary Material for “The volatilization behavior of typical fluorine-containing slag in steelmaking”

Reviewer comments

## Data accessibility

The datasets supporting this article have been uploaded as part of the electronic supplementary material.
