# Supplementary material for: The volatilization behaviour of typical fluorine-containing slag in steelmaking
Source: R Soc Open Sci. 2020 Aug 26;7(8):200704. doi: 10.1098/rsos.200704 (PMC7481700; doi:10.1098/rsos.200704)
Supplement: Supplementary Material for “The volatilization behavior of typical fluorine-containing slag in steelmaking” [file rsos200704supp1.pdf]

Supplementary Material for "The volatilization behavior of typical fluorine-containing slag in steelmaking"

Zhongyu ZHAO, Junxue ZHAO*, Zexin TAN, Boqiao QU, Yaru CUI

School of Metallurgical Engineering, Xi'an University of Architecture and Technology, Xi'an, China, 710055

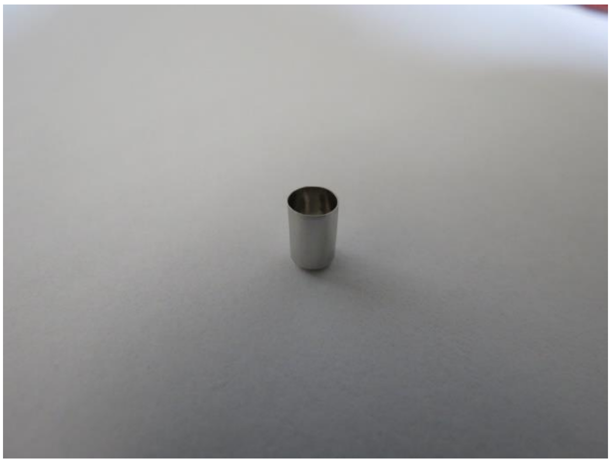

Fig. S1. Pt- Rh crucible for TG test

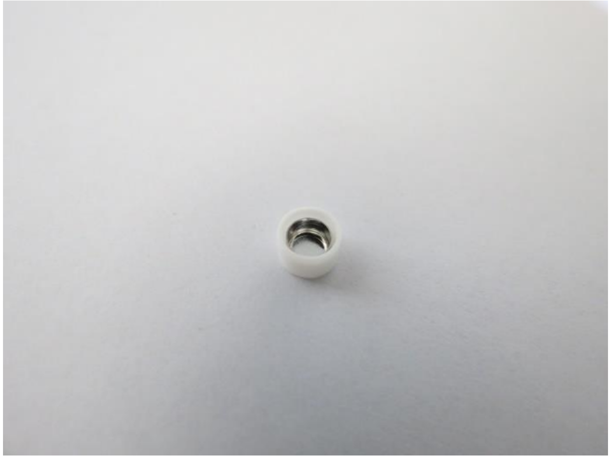

Fig. S2. Pt- Rh crucible for MS test

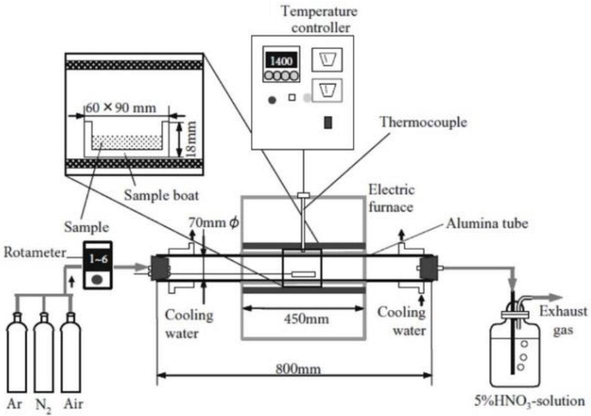

Fig. S3. Tubular furnace for roasting test
